# Risk Factors for Necrotizing Enterocolitis in Infants With Patent Arterial Duct. A Retrospective Matched Paired Analysis

**DOI:** 10.3389/fped.2020.00179

**Published:** 2020-04-28

**Authors:** Stephanie Haefeli, Marcin Kordasz, Catherine Tsai, Eva-Maria Hau, Peter Klimek, Dietmar Cholewa, Mladen Pavlovic, Steffen Berger, Ulf Kessler

**Affiliations:** ^1^Department of Pediatric Surgery, Inselspital, Bern University Hospital, University of Bern, Bern, Switzerland; ^2^Department of Pediatrics, Inselspital, Bern University Hospital, University of Bern, Bern, Switzerland; ^3^Department of Pediatric Surgery, Cantonal Hospital Aarau, Aarau, Switzerland; ^4^Department of Pediatrics, Cantonal Hospital of Fribourg, Fribourg, Switzerland; ^5^Center of Visceral Surgery, Klinik Beau-Site, Hirslanden, Bern, Switzerland

**Keywords:** necrotizing enterocolitis, NEC, patent ductus arteriosus, PDA, outcome, mortality, risk factors

## Abstract

**Background:** The development of necrotizing enterocolitis (NEC) in neonates with patent ductus arteriosus (PDA) is not well-understood. Our aim was to find risk factors for NEC in children with a significant PDA and to assess differences in mortality and duration of hospital stay between patients with PDA and those with PDA and NEC.

**Methods:** We performed a retrospective single center case control study including infants with PDA scheduled for treatment. We compared multiple patient data between patients with PDA and those with PDA and NEC from 2004 to 2018 using 1:2 and 1:1 matching.

**Results:** We used 1:2 matching with 26 NEC patients (cases) and 52 PDA patients without NEC (controls) and 1:1 matching with 5 NEC patients and 5 PDA patients without NEC. NEC patients had lower Apgar score (1′), more congenital malformations, more suspected sepsis, less hypotension, higher minimum platelet count and higher CRP-values during the week before NEC (*P* < 0.05, respectively). The mortality was higher in NEC cases [29% (9/31)] compared to the control patients [2% (1/57), *P* < 0.001]. Lower Apgar score (1′) was correlated with an increased risk of NEC stage III. Hypotension was inversely correlated with the odds of NEC (OR 0.3).

**Conclusions:** NEC increased mortality in infants with PDA. Hypotension did not increase the risk of NEC in infants with PDA. Routine clinical parameters were not able to predict NEC in infants who suffer from PDA.

## 1. Introduction

Necrotizing enterocolitis (NEC) affects up to 7% of preterm neonates below 1,500 g of birth weight (1). Additionally, preterm infants also frequently suffer from patent ductus arteriosus (PDA) (2). Disturbance of intestinal microcirculation is one of the factors that contributes to necrosis of the gut in NEC. Accordingly, hypotension has been found to be a risk factor for NEC (3, 4). Since PDA affects blood flow, it is believed, that hemodynamically relevant PDA might precipitate NEC (2).

In addition to PDA itself, PDA-related treatment might also play a role in the pathophysiology of NEC in infants with PDA. In a metaanalysis by Ohlsson et al. (5) NEC occurred significantly more often in patients treated for PDA with indomethacin as compared to infants treated with ibuprofen.

Taken all together, it remains largely unknown—and impossible to predict—which neonates with PDA will develop NEC.

Our primary aim was to find risk factors for NEC in children with a significant PDA. Our secondary aim was to assess differences in outcomes (duration of hospital stay and mortality) between the two groups of patients (PDA vs. PDA and NEC).

## 2. Methods

We performed a single-center retrospective case-control study including patients who were born between December 2004 and June 2018 and treated at the University Children's Hospital in Bern, Switzerland. During this period 176 newborns were diagnosed with NEC in our hospital. Our study was approved by the local ethics committee (Kantonale Ethikkomission Bern, Basec-Nr.: 2016-00477).

Our inclusion criteria for cases were defined as development of NEC (Bell stage ≥ 2) (6) after being diagnosed with PDA. We only included patients with a PDA who were scheduled for treatment. On an intention-to-treat basis we also included patients in whom there was an indication for treatment, however they never received the treatment (for example due to low platelet count or other clinical factors). We performed Bell staging based on a patient chart review, including radiographic imaging. We included patients with all gestational ages. We excluded infants with a PDA who were not scheduled for treatment.

For each index case suffering from PDA and NEC, we matched two controls that each had a PDA without NEC. We used the following matching criteria: (1) birth weight (BW): if the BW of the index patient was <1,000 g, we accepted a maximum difference of 100 g between the index and the control patient; if the BW was 1,000–1,999 g, we accepted a maximum difference of 200 g; if the BW was 2,000–2,999 g, we accepted a maximum difference of 300 g; if the BW was >3,000 g, we accepted a maximum difference of 500 g. (2) gestational age (GA): We accepted a maximum difference of 2 weeks. (3) post-natal age at the time of indication for treatment of the PDA: we accepted a maximum difference of 5 days.

We extracted the following variables as potential risk factors for NEC from the patient charts: maternal age, maternal hypertension or preeclampsia or eclampsia, tocolysis during pregnancy, prenatal steroids, delivery mode, Apgar scores, congenital malformations/syndromes, surfactant treatment, delayed meconium passage, suspected sepsis—as defined by the presence of three of the following five criteria: elevated C-Reactive Protein (CRP), leucocytosis or leucopenia (we used the following reference values for first month of life: 5–25 G/L, later on: 3–15 G/L), elevated count of premature granulocytes, temperature instability and symptoms of infection (such as vomiting, diarrhea, irritabilty or neurological changes), hypotension [defined as a period of mean blood pressure below GA in weeks (7)], umbilical catheters, cardiac surgery, other surgery, ventilation (mechanical, Continuous Positive Airway Pressure), phototherapy, blood transfusion (erythrocytes, thrombocytes, fresh frozen plasma), drugs (indomethacin, systemic steroids, erythropoietin, catecholamines, morphine, antacid agents, immunoglobulins), nutrition (enteral feeding and parenteral feeding), antibiotic treatment before NEC onset (days of treatment and name of antibiotic agent), laboratory findings before NEC onset (umbilical artery/vein pH, minimal and maximal hemoglobin, minimal and maximal white blood cell count, minimal platelets, maximal lactate, minimal pH value, maximal CRP value), mortality, and duration of hospitalization. In most of these variables we extracted data for the overall time before NEC onset and the last 7 days before NEC onset. We extracted laboratory data for the controls in the same time span as for the cases (e.g., if the case developed NEC at the age of 10 days, we extracted the data up to this age for the case and as well for the matched controls).

The data were collected in the RedCap database (8) and analyzed using a combined script written in Perl (9), Python (10), and R (11). The descriptive analysis was followed by assessing the statistical differences between both groups. This was performed using the Wilcoxon signed rank test with continuity correction for continuous variables and McNemar's Chi-squared test for discrete variables. We considered results as statistically significantly if *P* was <0.05. Variables which yielded a statistically significant difference (as indicated in the Results section) were then analyzed using multi- and univariate logistic regression (including stepwise regression) for their influence on development of NEC, development of NEC stage III, mortality, and duration of hospitalization. For this purpose, the hospitalization duration was transformed into a binary variable: long hospital stay (longer than the median of 86 days or the third quartile of 114 days) and short hospital stay (shorter than the median or third quartile of the study population).

Continuous variables are presented as median and 95% confidence interval (CI95). Discrete variables have been approximated to binary variables (yes or no) and are presented as number of positive responses (“yes”) per total number of cases or controls and the percentage of positive responses. Whenever data for specific variables were unavailable, this particular case or control was excluded from the analysis of this variable.

## 3. Results

In the analysis, we included 31 patients with NEC and PDA and 57 controls with PDA alone. A 1:2 matching on 26 NEC cases and 52 controls was used, 5 remaining cases were matched 1:1 with 5 controls.

The initial study cohort consisted of 32 cases and 64 controls, but five of the included controls did not fulfill all of the matching criteria.

These five patients were included in the primary analysis, but due to high heterogeneity, they were excluded and for their matched cases a 1:1 matching was conducted. Additionally, in order to further reduce the heterogeneity, one outlier in terms of gestational age (31 6/7w) was excluded from the NEC group, together with its corresponding controls.

**Figure 1:**
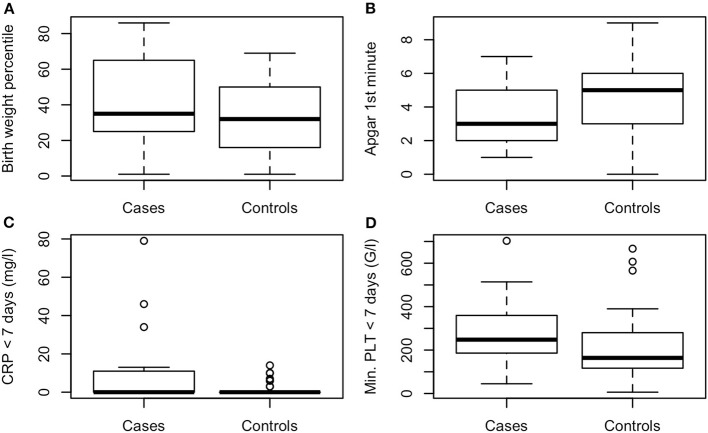
**(A)** Birth weight percentile, *P* < 0.001, **(B)** Apgar 1st minute, *P* < 0.01, **(C)** maximal CRP < 7 days before NEC onset (mg/l), *P* < 0.05, **(D)** minimal platelet count < 7 days before NEC onset (G/l), *P* < 0.01.

**Figure 2:**
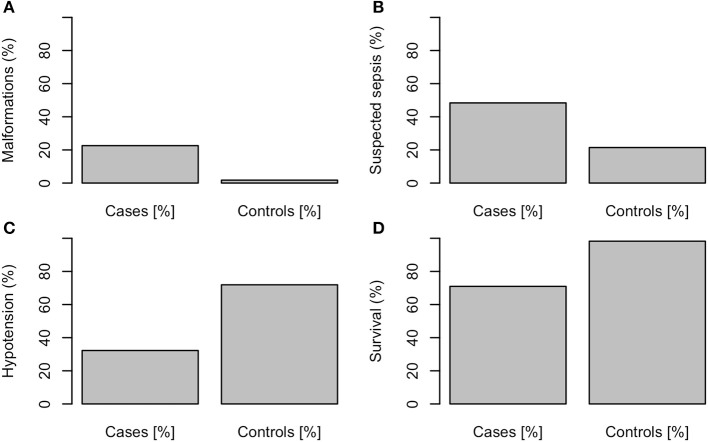
**(A)** Malformations, *P* < 0.001, **(B)** suspected sepsis, *P* < 0.01, **(C)** hypotension, *P* < 0.001, **(D)** survival, *P* < 0.001.

The following variables differed significantly between the 31 NEC patients and the 57 controls: NEC-patients had significantly lower gestational age (median, CI95: Cases: 25.3, 23.5–30.4, Controls: 26.0, 24.1–31.2, *P* < 0.01, values in the entire cohort ranged from 23.5 to 31.6), higher birth weight percentile (median, CI95: Cases: 35, 1.8–84, Controls: 32, 3.4–67, *P* < 0.001, see Figure 1), lower Apgar score at 1′ (median, CI95: Cases: 3, 1–7, Controls: 5, 1–8, *P* < 0.01, see Figure 1), more malformations [Cases: 7/31 (23%), Controls: 1/57 (2%), *P* < 0.001, see Figure 2], more often suspected sepsis [Cases: 15/31 (48%), Controls: 12/56 (21%), *P* < 0.01, see Figure 2], lower incidence of hypotension [Cases: 10/31 (32%), Controls: 41/57 (72%), *P* < 0.001, see Figure 2], higher CRP-values during the week before the development of NEC (median, CI95: Cases: 0, 0–66.6, Controls: 0, 0–10, *P* < 0.05, see Figure 1) and higher minimum platelet count during the week before the NEC onset (median, CI95: Cases: 248, 48.9–580, Controls: 164, 31.8–601, *P* < 0.01, see Figure 1).

The malformations/syndromes in the NEC group included: partial cleft in the maxilla, subglottic stenosis, hypospadia (*n* = 2), plagiocephalus, inguinal hernia/hydrocoele testis, Pierre Robin sequence, and polycystic kidney disease. There was just one malformation in the control group: Antenatal Bartter syndrome type 1.

The mortality was higher in NEC cases [29% (9/31)] compared to the control patients [2% (1/57), P<0.001].

Indomethacin therapy did not differ significantly between the two groups [Cases: 27/31 (87%), Controls: 50/57 (88%), *P* = 1]. Likewise, therapy with antibiotics prior to the onset of NEC was not significantly different [Cases: 29/30 (97%), Controls: 50/56 (89%), *P* = 0.1]. Catecholamine treatment did not differ between the two groups either [Cases: 7/29 (24%), Controls: 12/54 (22%), *P* = 1].

In the logistic regression assessing NEC risk in PDA patients, hypotension was inversely (OR 0.31, CI95 0.091–1, *P* = 0.06) and suspected sepsis positively (OR 3.6, CI95 1.1–13, *P* < 0.05) correlated with the odds of NEC.

A stepwise regression yielded an optimal model for multivariate regression assessing the risk of NEC in PDA patients which involved the minimal platelet count one week before NEC onset (OR 6.1, CI95% 0.01–0.24–1.60–29.96, *p*-value < 0.05) and clinically suspected sepsis before onset of the disease (OR 5.2, CI95% 0.01–0.24–1.60–29.96, *p*-value < 0.05).

Lower Apgar score at the 1st minute was correlated with an increased risk of NEC stage III (OR 0.45, CI95 0.18–0.84, *P* < 0.05)—see Table 1 and Supplementary Tables.

**Table 1:** Risk of NEC and of NEC-outcomes.

**Variable**	**OR**	**CI95%**	***p*-Value**
**NEC risk factors - univariate model**			
Suspected sepsis before NEC onset	3.6	1.1–13	0.036
**NEC risk factors - multivariate**			
Platelet count	6.1	1.60–29.96	0.013
Suspected sepsis before NEC onset	5.2	1.39–22.84	0.019
**Risk factors for Bell III - univariate model**			
APGAR 1st minute	0.45	0.18–0.84	0.034

The birth weight percentile correlated with long hospitalization, but with an OR close to 1 (OR 0.98, CI95 0.94–1, *P* = 0.1).

None of the parameters significantly affected survival.

All basic cohort data, laboratory parameters and regression results are shown in Supplementary Tables.

## 4. Discussion

We found very few clinical parameters that were correlated with the occurrence of NEC in our cohort of neonates with PDA.

Low Apgar score at 1st minute, high CRP during the week before NEC onset, high minimum platelet count during the week before NEC, low prevalence of hypotension, high birth weight percentile and low gestational age were associated with NEC.

The parameters that were correlated with NEC in the logistic regression were the absence of hypotension, and higher minimum platelet count. The correlation between absence of hypotension and NEC is highly surprising, since, according to animal studies (12, 13), hypotension may reduce intestinal blood flow and therefore would favor intestinal necrosis. The fact that the presence of hypotension did not increase the risk of NEC might suggest that NEC in infants with PDA might not be correlated with impaired macrocirculation.

Correlation between suspected sepsis and NEC has already been described by Rose and Patel (14).

The higher CRP levels during the week preceding NEC onset as compared to the same post-natal time period in controls and its influence on the incidence of NEC (*P* < 0.05) might suggest a role of increased inflammation in the development of NEC. To the knowledge of the authors, there is no evidence for systemic inflammation as an indicator preceeding the onset of NEC. However, Luo et al. have shown that CRP level is an indicator of deterioration in NEC in small for gestational age newborns (15).

We previously described that lower platelet count correlated with non-survival in surgically treated NEC children (16). Our present results indicate that higher minimal platelet count is rather a risk factor for developing NEC. It is difficult to interpret this finding since no maximal platelet count has been documented during the study—we therefore think that speculating about the risk of thrombosis, which has already been implicated in the pathogenesis of NEC (17), would not be legitimate.

The lower Apgar scores in the first minute of life correlated with the development of NEC stage III (see Table 1). One could hypothesize that a brief post-natal ischemic hit might have exposed individuals to an increased risk of NEC later on. However, the Apgar scores at 5 and 10 min were not significantly different between groups. We therefore can not conclude that an isolated low Apgar score (1′) reflected a pathophysiologic phenomenon, but rather an incidental unrelated and not repeatable finding. Low Apgar score at 1st minute post-partum has already been proven not to be a valuable prognostic factor (18).

Blood transfusion rate did not correlate with the incidence of NEC and did not affect the survival of NEC patients, which speaks against the hypothesis that red blood cell transfusion increases the risk of NEC, especially in patients with PDA (19). However, the absolute number of transfusions in our cohort was too low to draw definitive conclusions.

Mitra et al. (20) analyzed side effects of treatment of PDA with Non-Steroidal Inflammatory Drugs (NSAID) and did not find higher rates of NEC in children with high oral doses of ibuprofen. Consequently, they suggested that the PDA itself would be a risk for NEC and not the treatment for it. We did not find any differences in the two groups regarding treatment with indomethacin or ibuprofen so we cannot support or reject this statement.

The birth weight percentile and gestational age differed significantly between groups, but this finding is due to our imperfect matching criteria which were rather widely chosen since it was not possible to gather an appropriate cohort otherwise.

Unfortunately we could not assess enteral feeding (21) as a widely discussed risk factor in a sufficient way in our study setting.

As we expected, the mortality rate was higher in the NEC group. However, we could unfortunately not show any significant risk factors for NEC-related mortality.

A limitation of this study is especially the small number of patients included and the single center retrospective setting. An imperfect matching of the patients is also a source of potential bias. Moreover, the case-control design as such does not allow to identify predictors, but rather correlation between parameters.

Additionally, since due to internal database limitations we could not assess the entire number of infants with a PDA or with a PDA that were scheduled for treatment or who developed NEC in the period studied, we could not provide details of how often the respective diseases occurred during the study period.

Nevertheless, we were able to indicate some parameters that correlated with the development of NEC in patients with PDA (such as suspected sepsis, CRP level, platelet count). Further studies, including prospective studies, are needed to clarify this more thoroughly.

## Data Availability Statement

All datasets generated for this study are included in the article/Supplementary Material.

## Author Contributions

UK, E-MH, MP, SH, and SB contributed to the conception and design of the work. UK, E-MH, CT, SH, and MK contributed to the acquisition and analysis of data. All authors contributed to the interpretation of data for the work. All authors drafted the work, revised it, approved the final version, and agreed to the publication.

## Conflict of Interest

The authors declare that the research was conducted in the absence of any commercial or financial relationships that could be construed as a potential conflict of interest.

## References

[1] Neu J Walker WA. Necrotizing enterocolitis. N Engl J Med. (2011) 364:255–64. 10.1056/NEJMra1005408 21247316 PMC3628622

[2] Benitz WE. Patent ductus arteriosus in preterm infants. Pediatrics. (2016) 137:e21053730. 10.1542/peds.2015-3730 26672023

[3] McElhinney DB Hedrick HL Bush DM Pereira GR Stafford PW Gaynor JW. Necrotizing enterocolitis in neonates with congenital heart disease: risk factors and outcomes. Pediatrics. (2000) 106:1080–7. 10.1542/peds.106.5.1080 11061778

[4] Samuels N van de Graaf RA de Jonge RCJ Reiss IKM Vermeulen MJ. Risk factors for necrotizing enterocolitis in neonates: a systematic review of prognostic studies. BMC Pediatr. (2017) 17:105. 10.1186/s12887-017-0847-3 28410573 PMC5391569

[5] Ohlsson A Walia R Shah SS Ibuprofen for the treatment of patent ductus arteriosus in preterm or low birth weight (or both) infants. Cochrane Database Syst Rev. (2015) CD003481. 10.1002/14651858.CD003481.pub6 25692606

[6] Walsh MC Kliegman RM. Necrotizing enterocolitis: treatment based on staging criteria. Pediatr Clin North Am. (1986) 33:179–201. 10.1016/S0031-3955(16)34975-6 3081865 PMC7131118

[7] Dempsey EM. What should we do about low blood pressure in preterm infants. Neonatology. (2017) 111:402–7. 10.1159/000460603 28538235

[8] Harris PA Taylor R Thielke R Payne J Gonzalez N Conde JG. Research electronic data capture (REDCap)-a metadata-driven methodology and workflow process for providing translational research informatics support. J Biomed Inform. (2009) 42:377–81. 10.1016/j.jbi.2008.08.010 18929686 PMC2700030

[9] Wall L Christiansen T Orwant J Programming Perl. Sebastopol, CA: O'Reilly Media, Inc (2000).

[10] Python Software Foundation Python Language Reference, version 2.7. Available online at: http://www.python.org

[11] R Core Team R Foundation for Statistical Computing. Vienna (2017). Available online at: https://www.R-project.org/

[12] Cassuto J Cedgård S Haglund U Redfors S Lundgren O. Intramural blood flows and flow distribution in the feline small intestine during arterial hypotension. Acta Physiol Scand. (1979) 106:335–42. 10.1111/j.1748-1716.1979.tb06407.x 506768

[13] Redfors S Hallbäck DA Haglund U Jodal M Lundgren O. Blood flow distribution, villous tissue osmolality and fluid and electrolyte transport in the cat small intestine during regional hypotension. Acta Physiol Scand. (1984) 121:193–209. 10.1111/j.1748-1716.1984.tb07448.x 6475548

[14] Rose AT Patel RM. A critical analysis of risk factors for necrotizing enterocolitis. Semin Fetal Neonat Med. (2018) 23:374–9. 10.1016/j.siny.2018.07.005 30115546 PMC6269219

[15] Luo L Dong W Zhang L Zhai X Li Q Lei X. Correlative factors of the deterioration of necrotizing enterocolitis in small for gestational age newborns. Sci Rep. (2018) 8:13. 10.1038/s41598-017-18467-8 29311572 PMC5758570

[16] Kessler U Mungnirandr A Nelle M Nimmo AF Zachariou Z Berger S. A simple presurgical necrotizing enterocolitis-mortality scoring system. J Perinatol. (2006) 26:764–8. 10.1038/sj.jp.7211613 17122786

[17] Joshi VV Draper DA Bates RD. Neonatal necrotizing enterocolitis. Occurrence secondary to thrombosis of abdominal aorta following umbilical arterial catheterization. Archiv Pathol. (1975) 99:540–3. 1191123

[18] American Academy of Pediatrics The Apgar score. Pediatrics. (2006) 117:1444–7. 10.1542/peds.2006-0325 16585348

[19] Mohamed A Shah PS. Transfusion associated necrotizing enterocolitis: a meta-analysis of observational data. Pediatrics. (2012) 129:529–40. 10.1542/peds.2011-2872 22351894

[20] Mitra S Florez ID Tamayo ME Mbuagbaw L Vanniyasingam T Veroniki AA. Association of placebo, indomethacin, ibuprofen, and acetaminophen with closure of hemodynamically significant patent ductus arteriosus in preterm infants: a systematic review and meta-analysis. JAMA. (2018) 319:1221–38. 10.1001/jama.2018.1896 29584842 PMC5885871

[21] Berseth CL. Feeding strategies and necrotizing enterocolitis. Curr Opin Pediatr. (2005) 17:170–3. 10.1097/01.mop.0000150566.50580.26 15800406

