# Supplementary material for: Risk Factors for Necrotizing Enterocolitis in Infants With Patent Arterial Duct. A Retrospective Matched Paired Analysis
Source: Front Pediatr. 2020 Apr 28;8:179. doi: 10.3389/fped.2020.00179 (PMC7198791; doi:10.3389/fped.2020.00179)
Supplement: Supplementary file 1 [file Data_Sheet_1.PDF]

Supplementary Material

1 SUPPLEMENTARY TABLES

Table S1. Baseline cohort data

Value	Cases	Controls	p-value
Male gender	16/31 (52%)	28/57 (49%)	0.73
Born in the tertiary center	28/29 (97%)	52/53 (98%)	1
Year of birth	2010 (2005 - 2016)	2012 (2008 - 2017)	0.18
Non-Swiss nationality at birth	7/27 (26%)	14/50 (28%)	1
Birth weight (g)	720 (510 - 1564)	780 (494 - 1538)	0.4
Birth weight percentile	35 (1.8 - 84)	32 (3.4 - 67)	<0.001 (***)
Birth length (cm)	33 (29.5 - 42.2)	34.5 (29 - 42.8)	0.62
Birth length percentile	49 (4.5 - 85)	48 (7.2 - 83)	0.32
Birth head circumference (cm)	24 (21 - 30)	24 (20.7 - 29.8)	0.71
Birth head circumference percentile	47 (7 - 78)	35 (5 - 74)	0.21
Gestational age (weeks/days)	25.3 (23.5 - 30.4)	26.0 (24.1 - 31.2)	0.0034 (**)
Age of mother at birth of child (years)	30.8 (23.7 - 42.8)	29 (22.4 - 40.9)	0.34
Maternal preeclampsia/eclampsia	7/25 (28%)	7/45 (16%)	0.18
Tocolysis during pregnancy	25/27 (93%)	42/48 (88%)	0.45
Prenatal steroids	28/28 (100%)	50/52 (96%)	0.5
Number of infants in this birth	1 (1 - 3)	1 (1 - 4)	0.1
Assisted delivery	25/31 (81%)	47/57 (82%)	0.8
Apgar 1st minute	3 (1 - 7)	5 (1 - 8)	0.0039 (**)
Apgar 5 minutes	7 (2 - 9)	7 (3.38 - 9)	0.69
Apgar 10 minutes	8 (4 - 9)	8 (5 - 9.62)	0.98
Surfactant treatment	24/30 (80%)	48/55 (87%)	0.18
Congenital malformations or syndroms	7/31 (23%)	1/57 (2%)	<0.001 (***)
Delayed meconium discharge	2/7 (29%)	1/14 (7%)	0.38
Suspected sepsis before NEC onset	15/31 (48%)	12/56 (21%)	0.0059 (**)
Hypotension before NEC onset	10/31 (32%)	41/57 (72%)	<0.001 (***)
Umbilical catheter arteriel	11/29 (38%)	21/54 (39%)	0.85
Umbilical catheter venous	14/29 (48%)	29/54 (54%)	0.57
Cardiac surgery before NEC onset	3/31 (10%)	8/57 (14%)	0.55
Days of mechanical ventilation before NEC	2 (0 - 25)	2 (0 - 19.6)	0.51
Days of CPAP before NEC onset	9 (0 - 27.2)	7 (0 - 26.2)	0.91
Phototherapy before NEC onset	9/10 (90%)	18/19 (95%)	1
EC transfusion before NEC onset	7/30 (23%)	13/55 (24%)	1
EC transfusion <7 days before NEC onset	5/11 (45%)	6/16 (38%)	1
Platelets transfusion before NEC onset	1/29 (3%)	4/53 (8%)	0.25
FFP transfusion before NEC onset	3/29 (10%)	11/53 (21%)	0.21
Indomethacin therapy before NEC onset	27/31 (87%)	50/57 (88%)	1
Indomethacin therapy <7 days before NEC onset	15/25 (60%)	19/41 (46%)	0.26
Ibuprofen therapy before NEC onset	7/31 (23%)	7/57 (12%)	0.24
Systemic steroids before NEC onset	12/27 (44%)	16/49 (33%)	0.26
Systemic steroids <7 days before NEC onset	1/6 (17%)	3/8 (38%)	0.69

Table S2. Baseline cohort data

Value	Cases	Controls	p-value
Erythropoetin before NEC onset	21/28 (75%)	43/51 (84%)	0.11
Erythropoetin <7 days before NEC onset	16/18 (89%)	28/30 (93%)	0.62
Coffein before NEC onset	4/5 (80%)	9/9 (100%)	0.5
Catecholamine treatment before onset of NEC	7/29 (24%)	12/54 (22%)	1
Morphine infusion before onset of NEC	2/25 (8%)	4/44 (9%)	1
Antacid agents before NEC onset	2/27 (7%)	1/50 (2%)	0.5
Enteral feeding before NEC onset	3/6 (50%)	4/11 (36%)	1
Parenteral nutrition before NEC onset	29/29 (100%)	54/54 (100%)	1
Antibiotics before NEC onset	29/30 (97%)	50/56 (89%)	0.12
Days of (any) antibiotics before NEC onset	7 (2.18 - 22)	7 (1.23 - 19.8)	0.095 (.)
Penicilline	26/27 (96%)	44/50 (88%)	0.062 (.)
Cephalosporine	3/27 (11%)	10/50 (20%)	0.15
Carbapeneme	2/27 (7%)	1/50 (2%)	0.38
Aminoglycoside	25/27 (93%)	45/50 (90%)	1
Macrolide	2/27 (7%)	1/50 (2%)	0.25
Glycopeptide antibiotics	1/27 (4%)	1/50 (2%)	1
Survival	22/31 (71%)	56/57 (98%)	<0.001 (***)
Duration of hospitalisation (in days)	86.5 (33.8 - 211)	91.5 (44 - 143)	0.4
Umbilical artery pH	7.32 (7.2 - 7.43)	7.32 (7.11 - 7.44)	0.096 (.)
Umbilical venous pH	7.39 (7.33 - 7.51)	7.35 (7.29 - 7.39)	0.5
Min. Hb before NEC onset (g/l)	122 (91.4 - 172)	120 (90 - 158)	0.25

Table S3. Laboratory parameters

Value	Cases	Controls	p-value
Max. Hb before NEC onset (g/l)	184 (152 - 214)	180 (151 - 227)	0.98
Min. Hb <1 week before NEC onset (g/l)	127 (92.5 - 179)	124 (92 - 158)	0.57
Min. Hb <2 weeks and $\geq$ 1 week (g/l)	133 (98.2 - 183)	132 (99.2 - 169)	0.51
Min. WBC <7 days before NEC onset (G/l)	11.2 (3.75 - 38.7)	11.4 (2.73 - 42.2)	0.087 (.)
Min. WBC before NEC onset (G/l)	6 (3.13 - 20.2)	6.73 (2.62 - 34.8)	0.95
Max. WBC (<1week before NEC onset) (G/l)	18.9 (8.13 - 50.3)	20.4 (8.76 - 52.4)	0.84
Max. WBC before NEC onset (G/l)	18.1 (10.8 - 48.5)	23.3 (9.35 - 62.5)	0.79
Min. platelets before NEC onset (G/l)	137 (48 - 270)	121 (30 - 244)	0.081 (.)
Min. platelets <7 days before NEC onset (G/l)	248 (48.9 - 580)	164 (31.8 - 601)	0.0092 (**)
Max. lactate before NEC onset (mmol/l)	3.3 (1.7 - 7.45)	4.5 (1.94 - 9.64)	0.072 (.)
Max. lactate <7 days before NEC onset (mmol/l)	2.4 (1.15 - 5)	2.1 (1.14 - 6.56)	0.48
Min. pH value before NEC onset	7.17 (6.99 - 7.3)	7.17 (7 - 7.26)	0.83
Min. pH value <7 days before NEC onset	7.25 (7.06 - 7.37)	7.24 (7.07 - 7.38)	0.21
Max. CRP value before NEC onset (mg/l)	3.5 (0 - 65)	0 (0 - 23.2)	0.18
Max. CRP value <7 days before NEC onset (mg/l)	0 (0 - 66.6)	0 (0 - 10)	0.032 (*)

2 ABBREVIATIONS USED

CPAP - Continuous Positive Airway Pressure
CRP - C-Reactive Protein
EC - Erythrocyte Concentrate
FFP - Fresh Frozen Plasma
Hb - Haemoglobine
Min. - Minimum
Max. - Maximum
NEC - Necrotizing Enterocolitis
WBC - White Blood Cell count

Table S4. Risk of NEC and of NEC-outcomes

Variable	OR	CI95%	p-value
**NEC risk factors**			
APGAR 1st minute	0.88	0.64 - 1.2	0.42
Gestational age	1	0.98 - 1.1	0.24
CRP <7 days	1.1	1 - 1.3	0.084 (.)
pH <7 days	6	0.009 - 1.2e+04	0.61
Birth weight percentile	1	0.99 - 1	0.12
Platelet count	1	1 - 1	0.13
Hypotension	0.31	0.091 - 1	0.055 (.)
Congenital malformations or syndroms	4.7e+07	0 - N/A	0.99
Suspected sepsis before NEC onset	3.6	1.1 - 13	0.036 (*)
**Risk factors for Bell III**			
APGAR 1st minute	0.45	0.18 - 0.84	0.034 (*)
Gestational age	0.96	0.88 - 1	0.27
CRP <7 days	0.97	N/A - 1	0.64
pH <7 days	4.1	0 - 1.1e+07	0.83
Birth weight percentile	0.97	0.93 - 1	0.14
Platelet count	1	1 - 1	0.59
Hypotension	0.62	0.079 - 3.5	0.61
Congenital malformations or syndroms	1.2	0.14 - 7.4	0.85
Suspected sepsis before NEC onset	0.55	0.094 - 2.8	0.48
**Survival**			
APGAR 1st minute	1.6	0.97 - 3	0.097 (.)
Gestational age	1.1	1 - 1.2	0.078 (.)
CRP <7 days	0.98	0.93 - 1	0.48
pH <7 days	330	0.006 - 1e+08	0.31
Birth weight percentile	1	0.99 - 1.1	0.14
Platelet count	1	0.99 - 1	0.84
Hypotension	0.47	0.09 - 2.4	0.36
Congenital malformations or syndroms	3	0.41 - 62	0.35
Suspected sepsis before NEC onset	0.67	0.13 - 3.2	0.61
**Long hospitalisation - median**			
APGAR 1st minute	1	0.68 - 1.6	0.88
Gestational age	1	0.96 - 1.1	0.61
CRP <7 days	0.99	0.94 - 1	0.81
pH <7 days	0.0037	0 - 44	0.26
Birth weight percentile	0.99	0.96 - 1	0.56
Platelet count	1	0.99 - 1	0.82
Hypotension	4.4	0.85 - 34	0.1
Congenital malformations or syndroms	0.95	0.17 - 5.7	0.96
Suspected sepsis before NEC onset	0.69	0.16 - 2.9	0.61
**Long hospitalisation - 3. quartile**			
APGAR 1st minute	1	0.66 - 1.6	0.94
Gestational age	1	0.98 - 1.1	0.28
CRP <7 days	0.99	0.93 - 1	0.82
pH <7 days	831	0.033 - 1.9e+08	0.22
Birth weight percentile	0.98	0.94 - 1	0.14
Platelet count	1	1 - 1	0.16
Hypotension	1.3	0.27 - 6.4	0.72
Congenital malformations or syndroms	1.5	0.25 - 8.5	0.64
Suspected sepsis before NEC onset	0.47	0.095 - 2.1	0.32
